# Pleiotropic effects on proliferation and mineralization of primary human adipose tissue-derived stromal cells induced by simvastatin

**DOI:** 10.1098/rsob.210337

**Published:** 2022-06-08

**Authors:** Martin Mariano Isabelo Sabandal, Edgar Schäfer, Simon Petsching, Susanne Jung, Johannes Kleinheinz, Sonja Sielker

**Affiliations:** ^1^ Central Interdisciplinary Ambulance in the School of Dentistry, University of Münster, Albert-Schweitzer-Campus 1, Gebäude W30, Waldeyerstr. 30, 48149 Münster, Germany; ^2^ Department of Cranio-Maxillofacial Surgery, University Hospital Münster, Münster, Germany

**Keywords:** adipose tissue-derived stromal cells, mineralization, pleiotropic effects, simvastatin

## Abstract

The circulating low-density lipoprotein concentration in blood can be reduced by the administration of statins. Frequently simvastatin (SV) is prescribed. Due to the reported pleiotropic effects of SV the aim of this study was to evaluate mineralization effects on human adipose tissue-derived stromal cells upon administration of SV. After informed consent human adipose tissue-derived stromal cells were obtained from tissue surplus of regular treatments of 14 individuals. According to established protocols after adding various SV concentrations (0.01 µM, 0.1 µM, 1.0 µM, 2.0 µM), alkaline phosphate (osteoblastic marker), mineralization capability and viability were determined at day 18, 21 and 28. The Kruskal–Wallis test was performed for statistical analysis. After adding SV a dose-dependent significant decreased viability and levels of alkaline phosphatase (*p* < 0.01) and a significantly increased mineralization (*p* < 0.01) of the primary cultures was recognized during the late mineralization stage. Mineralization of the human adipose tissue-derived stromal cells was induced by SV, possibly originated from alternative pathways than the alkaline phosphatase pathway. Further investigations should be performed regarding switching into the osteoblastic differentiation and as a possible source of cells that can be used as the basis for a potential bone graft substitute, which may allow an extension of the field of application.

## Graphical abstract

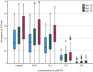

## 1. Introduction

To treat increased cholesterol and triglyceride concentrations in circulating blood the group of statins was developed as a therapeutic agent which reduces the concentration of circulating low-density lipoprotein (LDL) in blood [1]. Statins act as a successor of the fibric acid derivatives (fibrates), which were formerly used to reduce circulating triglycerides [2]. By contrast to previously used fibrates, statins can effectively reduce circulating blood concentrations of LDL [2]. As a member of the statins simvastatin (SV) is one of the first 3-hydroxy-3-methylglutaryl-CoA (HMG-CoA) reductase inhibitor [1]. Relevant findings prior to the administration of statins were especially hypercholesterolemia in association with increased risk of atherosclerosis related to the risk of cardiac infarction and additionally coronary heart disease [3]. In 2013, a recognized dose-dependent increased risk of rhabdomyolysis led to a reduction of the recommended daily maximum dose from 80 mg to 40 mg [4].

The target enzyme of SV is the HMG-CoA reductase. HMG-CoA reductase is located within the cholesterol biosynthesis and mevalonate pathway in which the enzyme can be reversible inhibited by SV. Inhibition decreases the intracellular concentration of mevalonate, which downregulates HMG-CoA reductase turnover in a feedback loop. Beside the direct inhibition the cellular LDL receptor expression is upregulated [3] the increased expression LDL receptor expression results in an increased LDL uptake from the circulating blood and an intravascular reduction of LDL concentration [3].

During the long-lasting administration of statins, so-called adverse effects have been recognized. Only few studies upon human cells or tissues investigated possible pleiotropic effects of statins. Pleiotropic effects were defined by different functions of for example genes which can show under different circumstances altering effects/functions. Also, drugs can show pleiotropic effects beside the targeted ones. The examined cells were adipose tissue cells [5–7], osteoblasts [8–10], odontoblast-like cells [11–13] and cells originated from the bone marrow [14]. Increased osteoblastic differentiation [14–16], increased viability and proliferation of osteoblasts [9,17] and improvement of the mineralization [18–20] have been reported as pleiotropic effects of SV.

Adult mesenchymal stem cells can be found in various tissues of the human body. The advantage of these cells is that they can differentiate into various other cell types and tissues under defined stimuli. Bone marrow-derived mesenchymal stromal cells (MSCs) were considered the optimal cells for regeneration of bone defects as the first choice. MSC can differentiate into different cell types within the limits of their original blastodermic layer. For personalized regenerative strategies it is necessary that stem cells can overcome the histological limitation of their initial embryonic germ layer and their capacity to differentiate into progenitor cells and ultimately into cell types of another origin [21]. Adipose tissue is a source of tissue-specific MSC human adipose tissue-derived stromal cells (hADSC). Adipose tissue can be found in various locations in the human body so it can be easily gained. In cell culture, the cells can be grown with a high proliferation rate without much effort [22]. Furthermore, these cells show stem cell characteristics across multiple passages and are thus an optimal source of multipotent stem cells [23,24]. In previous studies, our group has shown that hADSC isolated from human adipose tissue of different origins can easily transdifferentiate into an osteogenic lineage [25,26]. Compared to other sources of somatic stem cells, hADSC from fat tissue is a good cell source for autologous tissue regeneration. In two previously described studies with a broad group of human osteoblasts isolated from the jaw and human odontoblast-like cells isolated from the pulp, our group could demonstrate positive effects of SV upon mineralization and osteogenic differentiation [8,11]. The aim of this study was to evaluate mineralization effects on human adipose tissue-derived stromal cells upon administration of SV.

## 2. Results

### 2.1. Cell viability (MTT-assay)

**Figure 1.**
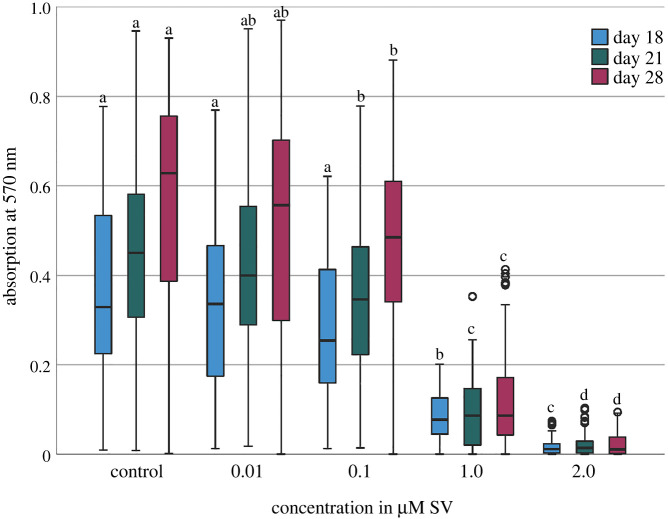
Viability given as photometric absorption at *λ* 570 nm of the MTT-assay. Different letters indicate statistically significant differences at *p* < 0.05; the upper and lower whiskers show the maximum and minimum values; the box represents 50% of the values and the horizontal line within the box displays the median value.

At each point of investigation, the viability of the hADSC with SV concentrations of 1.0 and 2.0 µM showed a significant decrease in cell viability (*p* < 0.01) compared to the groups with 0.01, 0.1 µM SV and the control group, with statistically significant differences between the groups with 2.0 µM and 1.0 µM SV (*p* < 0.01) (figure 1). At day 28 the group with 0.1 µM SV significantly differed from the control group (*p* < 0.05). Only slight differences (*p* > 0.05) of the viability values between the control group and the groups with 0.01 and 0.1 µM SV were recognizable (figure 1).

### 2.2. Osteogenic marker (ALP assay)

**Figure 2.**
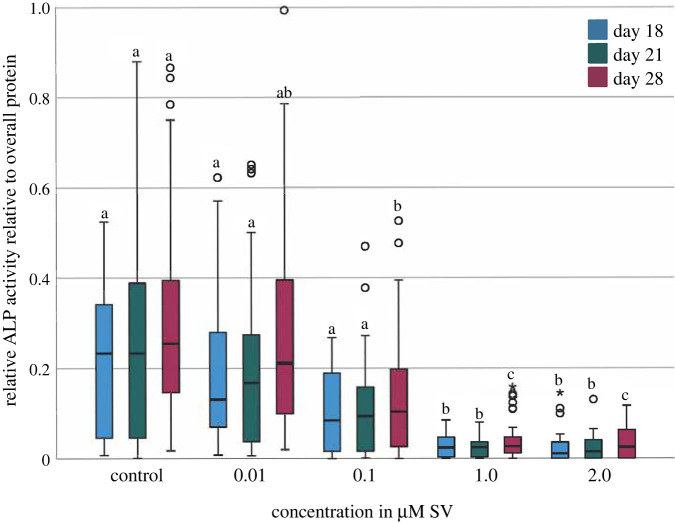
Relative activity of alkaline phosphatase (ALP) normalized to the overall protein. Different letters indicate statistically significant differences at *p* < 0.05; the upper and lower whiskers show the maximum and minimum values; the box represents 50% of the values and the horizontal line within the box displays the median value.

Especially groups with lower concentrations of 0.1, 0.01 µM SV and the control group showed extended 50% quartiles on each point of investigation of the relative activity of ALP. At each point of investigation the groups with 1.0 and 2.0 µM SV displayed a significant (*p* < 0.01) decrease in the relative activity compared with the groups with 0.1, 0.01 µM SV and the control group. At day 28 the group with 0.1 µM SV showed a significant decrease of the activity of ALP compared to the control group (figure 2).

### 2.3. Mineralization (alizarin red S staining)

**Figure 3.**
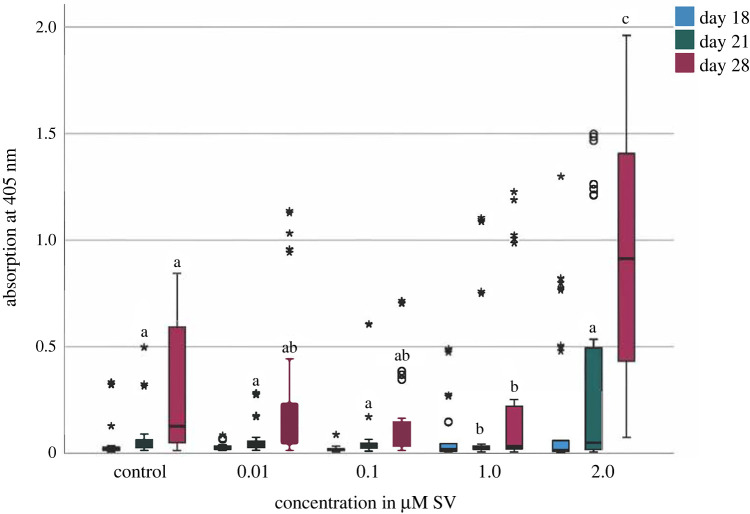
Mineralization given as photometric absorption at *λ* 405 nm of the alizarin red S staining. Different letters indicate statistically significant differences at *p* < 0.05; the upper and lower whiskers show the maximum and minimum values; the box represents 50% of the values and the horizontal line within the box displays the median value.

At day 18, no significant alterations of the mineralization was recognizable, while at day 21 the mineralization of the group with 1.0 µM SV was significantly (*p* < 0.05) decreased compared to the group with 0.1 µM SV. Additionally, at day 28 the mineralization of the group with 1 µM SV was significantly (*p* < 0.05) decreased compared to the control group, while the mineralization capability of the group with 2.0 µM SV was significantly (*p* < 0.01) increased compared to all other groups (figure 3). Only the group with 2.0 µM SV showed an earlier mineralization point.

## 3. Discussion

The current study is based on previous published studies investigating pleiotropic effects on primary human odontoblast-like cells and primary human osteoblasts under influence of SV [8,11]. These previous studies showed the necessary time of 9 [8] to 14 [11] days to induce mineralization. Additionally performed preliminary studies regarding die induction of osteoblastic differentiation of hADSC (data not shown) showed additionally needed time for osteoblastic differentiation up to 4 to 9 days. As published in the previous studies SV enhances differentiation and mineralization of such induced cells [8,11], so a relative short time between the first points of investigation is needed in order to evaluate the early alterations of the investigated protein levels like the activity of alkaline phosphatase relative to overall protein. In previous studies mineralization potential [6,27], expression of osteogenic markers on gene and protein level [10,14,27] and osteoblastic differentiation and proliferation [9,14,27] were investigated under influence of SV. The used human cells were, for instance, obtained from tissues like bone marrow and mesenchymal stem cells [14], periodontal ligament [10,27], odontoblast-like cells [11], cells from adipose tissues [5,28] and osteoblasts from bone chips [8]. The used SV concentrations in animal studies of rodent origin [6,7,15–20,29–33] were quiet equal.

hADSC is an easily obtainable tissue as a promising source of for bone tissue regeneration. Induction of osteogenic differentiation is possible according to standard scheme as in other tissues [25,26].

Little is known regarding osteogenic mineralization and differentiation effects of SV upon hADSC. Four studies investigating the effect of SV to ADSC of human and rodent origin [5–7,28], three of them evaluated the effects of SV impregnated scaffolds [6,7,9]. Two of these studies investigated the effect of SV on adipose tissue-derived cells in only one cell culture [6,7], the other studies contained the test results of three [28] and five cultures [5], respectively. Previously published studies investigating human osteoblasts [8] and human odontoblast-like cells [11] showed significant reduced cell viability with SV concentration of at least 1.0 µM and a significantly increased mineralization capability related to higher SV concentrations [8,11]. The design of this study is adapted to the design of previous studies [8,11] and confirms the previously reported results of reduced viability as well as a significantly increased mineralization capacity also for the hADSC by including a large number of cell cultures for this cell type.

During osteogenic differentiation, ALP is represented with a higher protein content and is synthesized in a greater extent as a typical osteogenic marker [18,34], which can also explain a higher turnover rate [35]. According to the recently described methods [10,27], the relative activity of ALP normalized to the total expressed protein was determined in the present study. The increased ALP turnover of mineralizing cells under the influence of SV may correlate with increased osteoblastic differentiation [14]. Such an increase in turnover of ALP seems to be usually time and dose dependent [27].

At each point of investigation the present study revealed significant decreases (*p* < 0.01) of the groups with 1.0 and 2.0 µM SV compared to all other groups. On the contrary, a significant increase of ALP within studies investigating SV impregnated scaffolds regarding the effect of SV determined at day 7 and 14, respectively [6,7,28], but only one of them investigated hADSC [28]. Previous findings shows observation periods up to 14 days so a comparison to the present results with an observation period of 28 days in the present study is limited [5–7,28]. In contrary to other published studies, in the present one the time of investigation was up to 28 days and not only up to 14 days to ensure reliable determination of possible alterations during osteoblastic differentiation of hADSC, having in mind that such cells have to change the differentiation pattern which requires additional time. Additionally, it takes some more time when evaluating alterations of protein levels as investigated in the present study. Furthermore, the use of scaffolds makes it impossible to trace the exact concentration of SV released from the scaffolds [6,7,28].

According to the methodology of other studies the cell viability after exposition to SV was performed within the present investigation [8,11]. The investigation period ranged up to 28 days, whereas similar studies investigated the cell viability from day 1 [7] up to 9 days [5]. By contrast to the findings of recent studies the cell viability was significantly (*p* < 0.05) reduced when adding 1.0 and 2.0 µM SV compared to the groups with 0.01 and 0.1 µM SV and the control. One study used SV supplements up to 2.0 µM which caused an increase of cell proliferation and viability [5], while a dose-dependent decrease in viability is reported by another study during an investigated during 24 h [7]. Thus, the results of the present and the study of Hajihasani Biouki *et al*. [7] appear to confirm a dose-dependent reduction in cell viability. Sabandal *et al*. found similar results in their study for a concentration of 1.0 µM SV when using odontoblast-like cells, so this could confirm a dose-dependent reduction in cell viability as well [11].

According to a previously published protocol the mineralization capability of cells with osteogenic differentiation was determined [8,11] by using alizarin red S staining [5,14,27]. The present results are in accordance with those of previous studies investigating hADSC. Three studies reported an increased but inconsistent mineralization capability at day 14 [5,6,28]. When investigating mineralization capability related pleiotropic effects of SV, often an increasing effect is determined in different cell types such as osteoblasts [8,10,14] and odontoblast-like cells [11–13]. In comparison to other studies, the increase in mineralization upon influence of SV appears to be delayed after induction of hADSC. Obviously, hADSC require more time to differentiate and mineralize after induction. This assumption is supported by the results of the current study, which showed increased mineralization at day 28. At the same point of time, there was also a significant (*p* < 0.01) increase of the mineralization of the group with 2.0 µM SV compared to all other groups (figure 3). In comparison, mineralization under the influence of SV in human osteoblasts and human odontoblast-like cells already occurred on day 16 and day 21, respectively [8,11].

As well as cells from the dental pulp hADSC can easily switched to an osteogenic differentiation. This induction can be accomplished by common media such as the combination of dexamethasone, ascorbic acid and ß-glycerophosphate added to the culture medium [28,29]. The aim of this study was to evaluate mineralization effects on human adipose tissue-derived stromal cells upon administration of SV. The investigated effects of SV upon mineralizing cells like induced hADSC can be defined as pleiotropic effects of SV. When the results of the current study were combined with the findings of other studies [5–7,28], hADSC appear to be a promising source of cells that can be used as the basis for a potential bone graft substitute.

## 4. Material and methods

The study evaluated effects of SV upon osteogenic and mineralization potential of 14 primary hADSC cultures originated from different donors. The obtained hADSC were collected in the context of previous studies [25,26,36]. The study was designed according to previous studies [8,11].

The conditions of the cell culture have already been described in previous studies [8,11,25,26]. Isolation and characterization of hADSC stem cells was performed by immunohistological determination of the expression of CD13, CD44 and CD90 according to previous published studies [25,26]. As a basal culturing medium, α-MEM (Lonza, Switzerland) supplemented with 10% bovine calf serum was used. For osteogenic differentiation, dexamethasone [16 ng ml^−1^], ascorbic acid [1.4 mM] and ß-glycerophosphate [10 mM] was added. SV was dissolved in ethanol_abs_ to a final stock solution of 6 mM stored at 4°C. Without prior activation of SV, various concentrations (0.01 µM, 0.1 µM, 1.0 µM and 2.0 µM) were prepared by diluting the stock solution of SV with culturing medium and mixed freshly. According to the dilution, the final concentration of ethanol was 0.1% in the culturing medium with 1.0 µM SV. Cells were seeded in a density of 4.000 cells/cm^2^ in 48-well plates (Greiner Bio-One) and allowed to adhere for 24 h before SV was added. As control group, cells with osteogenic induction and without SV in culturing medium were used. Cell viability, osteogenic activity and mineralization were analysed at day 18, 21 and 28. Cell culture was performed with three replicates. Cell viability was analysed with an in-house MTT assay and mineralization capability with a modified alizarin red S staining. Osteogenic activity was analysed using an alkaline phosphatase (ALP) assay and total protein was analysed with a Pierce BCA Protein Assay (Thermofisher Scientific), in which the protein content is determined by calorimetric measurement of Cu^1+^ ions by bicinchoninic acid (BCA). All assays have been described previously [8,11].

SPSS v. 27 (IBM, Ehningen, Germany) was used for statistical analysis. The sample values were assigned to the concentration groups on each point of investigation. No normal distribution of the data was present (according to the Kolmogorov–Smirnov test), thus the Kruskal–Wallis test was used. A comparison was done with the groups of the specific days. The level of significance was set at *p* < 0.05.

## 5. Conclusion

As published before, cells of different human tissues [8,11] can be influenced by SV regarding mineralization and osteogenic differentiation. The current study can confirm the influence of SV on hADSC and shows significant effects on mineralization (*p* < 0.01) in the 14 primary hADSC cultures investigated, especially at higher concentrations of SV. The present findings revealed a reduction of the cell viability and relative activity of ALP with an increase in mineralization induced by SV as a pleiotropic effect. Observed effects are time and dose-dependent effects of SV on hADSC, as shown before. The decreased cell viability (figure 1) with significantly (*p* < 0.01) decreased conversion of ALP (figure 2) and significantly (*p* < 0.01) increased mineralization when using higher SV concentrations. A more detailed investigation regarding the effect of SV on the metabolism of mineralization should be carried out for a more detailed understanding of divergent mineralization mechanisms to be able to evaluate potential clinical applications of SV.

## Data Availability

The supporting datasets were deposited in an external repository at https://doi.org/10.17879/04069454043 [37].
